# Monoterpenoid Indole Alkaloids from *Alstonia yunnanensis* and Their Cytotoxic and Anti-inflammatory Activities

**DOI:** 10.3390/molecules171113631

**Published:** 2012-11-16

**Authors:** Peng Cao, Yong Liang, Xu Gao, Xiao-Ming Li, Zhen-Quan Song, Guobiao Liang

**Affiliations:** Department of Neurosurgery, Institue of Neurology, General Hospital of Shenyang Military Area Command, Shenyang Northern Hospital, #83 Wenhua Road, Shenhe District, Shenyang 110018, China

**Keywords:** *Alstonia yunnanensis*, Apocynaceae, monoterpenoid indole alkaloids, cytotoxicity, anti-inflammatory activities

## Abstract

The 80% ethanol extract of *Alstonia yunnanensis* afforded five new monoterpenoid indole alkaloids: 11-hydroxy-6,7-epoxy-8-oxo-vincadifformine (**1**), 14-chloro-15-hydroxy-vincadifformine (**2**), perakine *N*_4_*-*oxide (**3**), raucaffrinoline *N*_4_*-*oxide (**4**), and vinorine *N*_1_,*N*_4_-dioxide (**5**), together with three known compounds: 11-methoxy-6,7-epoxy-8-oxo-vincadifformine (**6**), vinorine *N*_4_-oxide (**7**) and vinorine (**8**). The structures of the isolated compounds were established based on 1D and 2D (^1^H-^1^H-COSY, HMQC, HMBC, and ROESY) NMR spectroscopy, in addition to high resolution mass spectrometry. The isolated compounds were tested *in vitro* for cytotoxic potential against seven tumor cell lines and anti-inflammatory activities. Compounds **3**, **4** and **7** exhibited weak cytotoxicity against the tested cell lines and selective inhibition of Cox-2 (>85%).

## 1. Introduction

**Figure 1:**
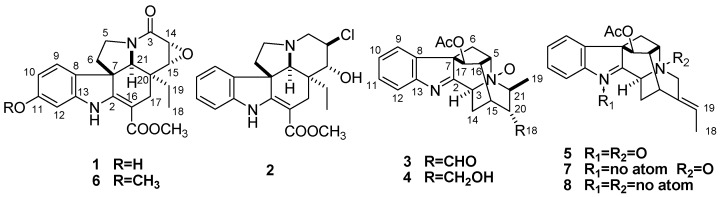
The structures of compounds **1**–**8**.

Plants of the genus *Alstonia* (Apocynaceae), which are usually shrubs or trees, are widely distributed throughout the tropical areas of the World, including Central America, Africa, Indo-Malaya, Australia and Asia [1,2]. They are mainly found in both lowland and highland forests, as well as in swampy areas [3]. The genus *Alstonia* comprises about 60 species, eight of which occur in China [4]. Several of these species are used in Traditional Medicine, for example in the treatment of malaria, dysentery, defervescence, antitussive, and to arrest hemorrhages [5,6,7,8,9,10]. Plants belonging to this genus represent a rich source of biologically-active unique heterocyclic alkaloids having a monoterpene indole skeleton [11,12,13,14,15,16,17,18,19,20,21,22,23,24,25]. Monoterpenoid indole alkaloids originate from the condensation of tryptophan with secologanin to give strictosidine and subsequent elaboration to give an impressive array of structural variants [26]. This type of alkaloids possess 19 (or 18) carbon atoms on the skeleton and reportedly have anticancer, antibacterial, antifertility, and anti-tussive activities [27,28,29,30]. *Alstonia yunnanensis* is a medicinal plant used for the treatment of fever, headaches, and inflammation in Southwest China [31]. We undertook a phytochemical investigation on the 80% ethanol extract of *A. yunnanensis*, resulting in the isolation of five new monoterpenoid indole alkaloids: 11-hydroxy-6,7-epoxy-8-oxovinca-difformine (**1**), 14-chloro-15-hydroxyvincadifformine (**2**), perakine *N*_4_*-*oxide (**3**), raucaffrinoline *N*_4_*-*oxide (**4**), and vinorine *N*_1_*,N*_4_-dioxide (**5**) (Figure 1), and three known compounds: 11-methoxy-6,7-epoxy-8-oxovincadifformine (**6**), vinorine *N*_4_-oxide (**7**) and vinorine (**8**) [32] (Figure 1). The structures of these compounds were elucidated mainly by NMR spectroscopic and mass spectroscopic methods. Furthermore, the cytotoxic and anti-inflammatory activities of compounds **1**–**8** were evaluated *in vitro*.

## 2. Results and Discussion

**Figure 2:**
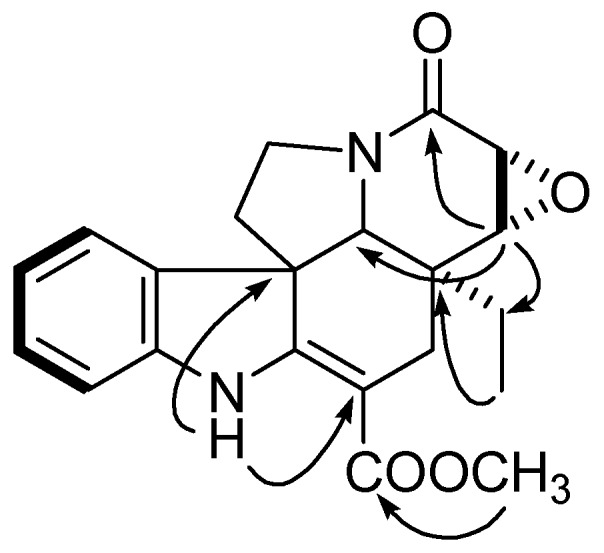
Key HMBC (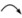) and ^1^H-^1^H-COSY (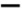) correlations of compound **1**.

Compound **1** was obtained as a colorless oil. Its positive HR-ESI-MS spectrum showed a quasimolecular ion peak at *m/z* 405.1423 [*M*+Na]^+^, consistent with the molecular formula C_21_H_22_N_2_O_5_ (calcd for C_21_H_22_N_2_O_5_Na 405.1426), accounting for 12 degrees of unsaturation. The IR spectrum exhibited absorptions at 3375, 1660 and 1620 cm^−1^ indicated the presence of a *β*-anilinoacrylate chromophore, corresponding to the carbon signals for an acrylate double bond at *δ*_C_ 166.5 (C-2) and 89.0 (C-16). The ^13^C-NMR and DEPT spectra of compound **1** displayed a substituted indole ring [*δ*_C_ 166.5 (s, C-2), 56.8 (s, C-7), 129.8 (s, C-8), 122.0 (d, C-9), 107.1 (d, C-10), 156.5 (s, C-11), 97.8 (d, C-12), 144.2 (s, C-13)] (Table 1). Besides the signals of the indole ring, the ^13^C-NMR spectrum displayed 13 additional carbon signals including four methine carbons (*δ*_C_ 22.3, 26.2, 42.1 and 43.6), three methylene carbons [two oxygenated (*δ*_C_ 51.2 and 57.1)], four quarternary carbons [two carbonyl (*δ*_C_ 165.0 and 168.3), one methoxy (*δ*_C_ 51.3), and one methyl group (*δ*_C_ 7.3). The ^1^H-NMR spectrum exhibited two characteristic proton signals for an oxirane ring at *δ*_H_ 3.62 and 3.48 (each, d, *J* = 4.0), three ABX-system protons at [*δ*_H_ 7.04 (d, *J =* 8.0 Hz, H-9), 6.33 (dd, *J =* 8.0, 2.0 Hz, H-10), 6.40 (d, *J =* 2.0 Hz, H-12)], and a NH signal at *δ*_H_ 8.96. The 14,15-epoxy group was established by the HMBC correlations of *δ*_H_ 3.48 (H-15) with *δ_C_* 165.0 (C-3), 26.2 (C-19) and 63.4 (C-21) and of *δ*_H_ 3.62 (H-14) with *δ_C_* 40.7 (C-20) (Figure 2). These data suggested that the structure of **1** was almost identical with **6** [32]. The distinct difference was that the methoxy at C-11 in **6** was replaced by an hydroxy group in **1**, which was supported by the observation of upfield chemical shift of C-11 from *δ_C_* 160.8 in **6** to *δ_C_* 156.5 in **1**. According to NOESY data and its negative specific rotation (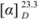 = −129.6), the *α*-orientation of the 14,15-epoxy group was determined by the correlation between H-15 and H-17*β*, which was possible only if the orientation of H-15 was *β* [32,33]. Thus, the structure of **1** was established as 11-hydroxy-6,7-epoxy-8-oxovincadifformine.

**Table 1:** ^13^C-NMR data of compounds **1**–**5** in CDCl_3_.

No.	1	2	3	4	5
2	166.5, s	166.6, s	179.7, s	180.3, s	147.4, s
3	165.0, s	54.6, t	74.7, d	74.6, d	68.8, d
5	43.6, t	51.0, t	67.0, d	66.9, d	67.6, d
6	42.1, t	44.3, t	33.9, t	33.8, t	33.5, t
7	56.8, s	54.5, s	65.5, s	65.5, s	58.4, s
8	129.8, s	137.9, s	136.3, s	136.5, s	132.9, s
9	122.0, d	121.3, d	125.6, d	125.6, d	126.2, d
10	107.1, d	120.4, d	127.9, d	127.8, d	131.0, d
11	156.5, s	127.5, d	130.4, d	130.4, d	1231.1, d
12	97.8, d	109.1, d	122.2, d	122.1, d	116.2, d
13	144.2, s	143.1, s	156.7, s	156.6, s	149.3, s
14	51.2, d	59.6, d	26.7, t	25.7, t	28.5, t
15	57.1, d	76.0, d	26.2, d	26.6, d	26.8, d
16	89.0, s	92.8, s	50.8, d	50.3, d	49.1, d
17	22.3, t	26.8, t	78.1, d	78.0, d	76.6, d
18	7.3, q	8.2, q	201.6, d	61.2, t	12.7, q
19	26.2, t	22.9, t	14.6, q	15.1, q	119.9, d
20	40.7, s	44.5, s	51.2, d	48.0, d	131.1, s
21	63.4, d	69.7, d	67.1, d	67.6, d	69.8, t
CO_2_*CH_3_*	51.3, q	51.2, q	20.8, q	20.2, q	20.9, q
*C*O_2_CH_3_	168.3, s	168.9, s	171.6, s	171.5, s	171.5, s

Compound **2** gave two isotopic peaks at *m/z* 388.1634 [M−1+H]^+^ and 390.1634 [M+1+H]^+^ in its HR-ESI-MS, accounting for a molecular formula of C_21_H_25_ClN_2_O_3_, suggesting 10 degrees of unsaturation. The existence of the chlorine atom was identified by the appropriate ^13^C-NMR chemical shift at *δ_C_* 59.6 (d, C-14) and EIMS analysis. The EI mass spectrometry showed one molecular and one quasi-molecular-ion peaks at *m/z* 387 (42.05, [*M*−1]^+^) and 389 (14.03, [*M*+1]^+^) with ratio of relative intensity approximating 3:1. The ^1^H-NMR spectrum exhibited four A_2_B_2_-system protons at *δ*_H_ 7.09 (d, *J =* 7.6 Hz, H-9), 6.80 (*dd*, *J =* 7.6, 1.8 Hz, H-10), 7.06 (dd, *J =* 7.6, 1.8 Hz, H-11), and 7.61 (*d*, *J =* 7.6 Hz, H-12), indicating the OH group at C-11 in **1** was absent in **2**. The NMR data were similar to those of compound **1**, except for a hydroxy group at C-15 (*δ_C_* 76.0) and a chlorine atom at C-14 (*δ_C_* 59.6) in **2** taking place the 14,15-epoxy group in **1** and **6**, as evidenced by the HMBC correlations and the ^1^H-^1^H-COSY correlations of proton signals at *δ*_H_ 4.20 (H-14) with *δ*_H_ 3.29 (H-3) and *δ*_H_ 3.92 (H-15). The *α*-orientation of H-14 and the *β*-orientation of H-15 were deduced from the NOESY correlations of H-14/H-21 and H-15/H-17*β*, respectively. Accordingly, compound **2** was identified as 14-chloro-15-hydroxyvincadifformine.

**Figure 3:**
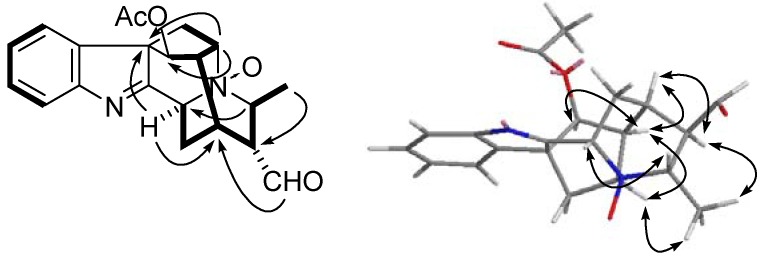
Key HMBC (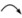), ^1^H-^1^H-COSY (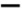), and NOESY (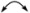) correlations of compound **3**.

Compound **3**, a colorless oil, exhibited a molecular formula of C_21_H_22_N_2_O_4_, based on the HRESIMS spectrum which showed a pseudomolecular ion at *m/z* 389.1475 [*M*+Na]^+^ (calcd. 389.1477). The UV absorptions at 220 and 264 nm showed the presence of an indolenine chromophore, which was confirmed by the characteristic chemical shift of C-2 at *δ*_C_ 179.7 in the ^13^C-NMR spectrum. The IR spectrum indicated the presence of aldehyde group (1725 cm^−^^1^), and benzene ring (1650 cm^−^^1^). The NMR data of **3** was almost identical with those of alstoyunine C [32] except for an aldehyde group in **3** instead of the carboxyl group at C-20 in alstoyunine C, which was confirmed by HMBC correlations of H-19 (*δ*_H_ 1.52, 3H, d, *J* = 6.6 Hz) with C-20 and H-18 (*δ*_H_ 9.84, 1H, *s*) with C-15 and C-21 respectively (Figure 3). The NOESY spectrum established the relative configuration of **3**. The correlations of H-5/H-19, H-3/H-21, and H-19/H-20 indicated the *α*-orientation of H-3, H-5, and H-21 and the *β*-orientation of H-20. In addition, the NOESY correlations of H-5/H-16, H-15/H-16, H-15/H-20, and H-16/H-17 suggested that the relative configuration of H-15, H-16, and H-17 were *α*-orientation. Therefore, compound **3** was elucidated as perakine *N*_4_*-*oxide.

Compound **4** was isolated as a colorless oil. The molecular formula C_21_H_24_N_2_O_4_ was established by a positive ion HRESIMS quasimolecular ion peak at *m/z* 391.1635 [*M*+Na]^+^. Comparing the NMR data of **4** with those of compound **3**, the only significant difference was that the aldehyde group was replaced by the signals of one hydroxymethyl group [*δ*_C_ 61.2; *δ*_H_ 3.37, 3.62 (each, 1H, m)]. The stereochemistry of **4** was expected to be the same as **3** on the basis of the NOESY data. Thus, compound **4** was elucidated as raucaffrinoline *N*_4_*-*oxide.

The molecular formula of the compound **5** was assigned as C_21_H_22_N_2_O_4_ on the basis of the quasimolecular ion peak [*M*+H]^+^ at *m/z* 367.1653 in the HR-ESI-MS. The 1D and 2D-NMR data were closely related to those of **7**. The only difference was compound **5** contains one more oxygen atom compared to **7**. The upfield chemical shift of C-2 from 178.1 in **7** to *δ*_C_ 147.4 in **5**, combined with the HMBC correlations of *δ*_H_ 5.15 (1H, *d*, *J* = 9.0 Hz, H-3), 2.10 (1H, dd, *J* = 14.8, 5.2 Hz, H-14a), and 2.43 (1H, dd, *J* = 14.8, 9.0 Hz, H-14b) with *δ*_C_ 147.4 (C-2), indicated that **5** was an *N*_1_-oxide derivative of **7**. The *E* configuration of the double bond between C-19 and C-20 was determined by the NOESY correlations of H-18 at *δ*_H_ 1.70 (3H, *d*, *J* = 7.2 Hz) with H-15 at *δ*_H_ 3.37 (1H, *t*, *J* = 5.2 Hz) and of H-19 at *δ*_H_ 5.45 (1H, q, *J* = 7.2 Hz) with H-21 at *δ*_H_ 4.03 (1H, d, *J* = 14.2 Hz). The NOESY analysis showed that the relative configurations of the C-3, C-5, C-15, C-16, and C-17 were in good agreement with those of compounds **3** and **4**. Therefore, compound **5** was identified as vinorine *N*_1_*,N*_4_-dioxide.

All these compounds were *in vitro* evaluated for their cytotoxic potential using BEN-MEN-1 (meningioma), CCF-STTG1 (astrocytoma), CHG-5 (glioma), SHG-44 (glioma), U251 (glioma), SK-MEL-2 (human skin cancer), and MCF-7 cells (human breast cancer). Alkaloids **3**, **4** and **7** exhibited cytotoxicity against all the tested tumor cell lines except BEN-MEN-1, while other compounds were noncytotoxic (IC_50_ > 50 μM). Furthermore, The IC_50_ value (Table 2) indicated that compound **3**, **4** and **7** possessed higher cytotoxic activities against astrocytoma and glioma cell (CCF-STTG1, CHG-5, SHG-44 and U251) with lower IC_50_ value than against human skin cancer (SK-MEL-2) and human breast cancer (MCF-7 cells).

**Table 2:** Cytotoxicity of compounds **1**–**7** against seven human tumor cell lines ^a^.

	Cell lines						
	BEN-MEN-1	CCF-STTG1	CHG-5	SHG-44	U251	SK-MEL-2	MCF-7
1	-	-	-	-	-	95.5	91.2
2	-	-	-	-	-	-	97.6
3	-	12.3	12.9	11.8	12.3	33.7	28.1
4	-	11.4	12.1	9.2	9.7	34.9	29.9
5	-	-	-	58.3	71.8	-	-
6	-	-	-	-	-	93.7	-
7	-	16.7	15.8	17.4	14.9	35.5	31.2
8	-	67.1	-	64.2	-	-	76.2
Adriamycin	17.8	24.7	21.8	33.7	28.4	37.6	14.1

^a^ Adriamycin are expressed as IC_50_ values in nM, and compound **1**–**7** are expressed as IC_50_ values in μM. (-) IC_50_ > 100 μM.

The compounds **1**–**8** were tested *in vitro* for their anti-inflammatory activities. The results of the anti-inflammatory assay were summarized in Table 3. Among the tested compounds, alkaloids **3**, **4** and **7** displayed selective inhibition of Cox-2 (>85%).

**Table 3:** Evaluation of Anti-Inflammatory Activity of Compounds **1**–**8** ^a^.

	COX-1	COX-2
**1**	27.35	<0
**2**	<0	<0
**3**	43.21	94.77
**4**	41.85	88.09
**5**	38.21	<0
**6**	36.98	<0
**7**	41.32	94.05
**8**	37.64	<0
SC-560	63.20	
NS-398		97.13

^a^ Percent inhibition (all compounds and reference drugs concentration: 100 μM).

Alkaloids **5** and **8** had no cytotoxic activities or selective inhibition of Cox-2 comparable to those of **3**, **4** and **7** although they possess the same monoterpene indole skeleton. The observations indicated that a *N*_4_-oxide functionality was essential for cytotoxic and anti-inflammatory properties, while a *N*_1_-oxide maybe weaken the cytotoxic and anti-inflammatory activities for this type of alkaloids.

## 3. Experimental

### 3.1. General

Optical rotations were measured on a Perkin-Elmer 341 polarimeter (Na filter, γ = 589 nm). UV spectra were obtained on a Shimadzu UV-2550 spectrophotometer, whereas IR spectra were recorded on a Perkin-Elmer 577 spectrometer with KBr disks. NMR spectra were measured on a Bruker AM-600 spectrometer. EIMS and HREIMS (70 eV) were carried out on a Finnigan MAT 95 mass spectrometer. All solvents used were of analytical grade (Shanghai Chemical Reagents Company Ltd., Shanghai, China). Silica gel (200–300 mesh), silica gel H (Qingdao Haiyang Chemical Co. Ltd., Qingdao ,China), C18 reversed-phase silica gel (150–200 mesh, Merck, New York, America), and MCI gel (CHP20P, 75–150 mm, Mitsubishi Chemical Industries Ltd., Tokyo, Japan) were used for column chromatography. HPLC separation was performed on an instrument consisting of a Waters 600 controller, a Waters 600 pump, and a Waters 2487 dual λ absorbance detector, with a Prevail (250 × 10 mm i.d.) preparative column packed with C18 (5 μm). Precoated thin-layer chromatography (TLC) plates with silica gel GF254 (Qingdao Haiyang Chemical Co. Ltd., Qingdao, China) were used for TLC.

### 3.2. Plant Material

The whole plants of *A. yunnanensis* were collected in Chuxiong, Yunnan Province, China, in September 2010. The sample was identified by one of the authors (G. B. Shi). A specimen (JGCS201009001) was deposited in the Herbarium of Shengyang Medicine College, Shengyang, China.

### 3.3. Extraction and Isolation

The dried whole plants of *A. yunnanensis* (15 kg) were extracted with 80% ethanol (40 L) three times under reflux for 15 h and then concentrated under reduced pressure to give a crude extract. The crude extract dissolved in 7% HCl (2 L) and filtered. The filtrate was made alkaline using 10% ammonia-water to pH 9–10. The basic solution was partitioned with EtOAc to give a total alkaloidal extract (42 g). The total alkaloidal extract was further fractionated on a silica gel column, eluted with CHCl_3_-Me_2_CO (from 1:0 to 0:1), to obtain eight fractions (1–10). Fraction 3 (petroleum ether-acetone 15:1, 2.3 g) was applied to an ODS MPLC column and eluted with MeOH-H_2_O (20:80, 30:70, 40:60, each 500 mL) to yield four subfractions (Fr. 3-1 and 3-4). Fr. 3-2 (MeOH-H_2_O, 310 mg) was purified by preparative RP-HPLC (ODS column, 250 × 20 mm) using MeOH/H_2_O (25:75) as mobile phase to obtain **6** (67 mg). Fr. 3-3 (MeOH-H_2_O, 330 mg) was chromatographed on a Sephadex LH-20 column eluted with MeOH/H_2_O (50:50), and purifed by preparative RP-HPLC (ODS column, 250 × 20 mm) using MeOH/H_2_O (34:66) as mobile phase to yield **1** (68 mg) and **2** (57 mg). Separation of fraction 5 (4.2 g) by silica gel column chromatography, eluted with petroleum ether-Me_2_CO (from 8:1 to 1:1), afforded six subfractions (Fr. 5-1 and Fr. 5-6). Fr. 5-2 (278 mg) was subjected to RP-18 (MeOH-H_2_O, from 2:8 to 6:4) and Sephadex LH-20 (MeOH) column chromatography to yield **7** (43 mg). Fr. 5-3 (MeOH-H_2_O 20:80, 303 mg) was repeatedly chromatographed on silica gel (chloroform-methanol gradient, from 20:1 to 10:1) and then purifed on a Sephadex LH-20 column eluted with MeOH/H_2_O (50:50) to afford **3** (78 mg). Fraction 8 (2.7 g) was subjected to Sephadex LH-20 (MeOH-H_2_O, 7:3) column chromatography to afford four subfractions (Fr. 8-1 and 8-4). Fr. 8-3 (120 mg) was purified by preparative RP-HPLC (ODS column, 250 × 20 mm) using MeOH/H_2_O (23:77 and 30:70, respectively) to afford **4** (44 mg) and **5** (52 mg), respectively.

*11-Hydroxy-6,7-epoxy-8-oxo-vincadifformine* (**1**). colorless oil. 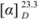
= −129.6 (*c* = 0.19, MeOH). UV (CDCl_3_) λ_max_(log *ε*) 325 (3.81), 245 (3.85), 228 (3.83), 197 (3.79) nm. IR (KBr) *ν*_max_ 3375, 1715, 1660, 1620, 1437, 1105, 755 cm^−1^. ^1^H-NMR (CDCl_3_, 600 MHz) and ^13^C-NMR (CDCl_3_, 125 MHz) data see Table 4 and Table 1 respectively. EI-MS *m/z*: 382 ([*M*]^+^). HR-ESI-MS (pos.) *m/z*: 405.1423 ([*M*+Na]^+^, C_21_H_22_N_2_O_5_Na. calc. 405.1426).

**Table 4:** ^1^H-NMR data of compounds **1**–**5** in CDCl_3_ (*δ* in ppm and *J* in Hz).

No.	1	2	3	4	5
N_1_-H	8.96 (s)	8.94 (s)	-	-	-
2	-		-	-	-
3	-	3.29 (m)	4.50 (d, 9.6)	4.48 (d, 9.6)	5.15 (d, 9.0)
4	-	-	-	-	-
5			4.28 (dd, 6.6, 5.0)	4.26 (dd, 6.6, 5.0)	4.25 (dd, 6.6, 5.2)
6a	3.21, 4.44 (m)	2.68, 2.98 (m)	2.45 (d, 12.8)	2.43 (d, 12.8)	2.58 (d, 12.8)
6b	1.74, 2.02 (m)	1.70, 2.11 (m)	2.89 (m)	2.87 (m)	2.89 (dd, 12.8, 4.5)
7	-	-	-	-	-
8	-	-	-	-	-
9	7.04 (d, 8.0)	7.09 (d, 7.6)	7.60 (d, 8.2)	7.57 (d, 8.2)	7.72 (d, 8.2)
10	6.33 (dd, 8.0, 2.0)	6.80 (dd, 7.6, 1.8)	7.29 (dd, 8.2, 1.8)	7.26 (dd, 8.2, 1.8)	7.56 (dd, 8.2, 2.0)
11	-	7.06 (dd, 7.6, 1.8)	7.42 (dd, 8.2, 1.8)	7.39 (dd, 8.2, 1.8)	7.61 (dd, 8.2, 2.0)
12	6.40 (d, 2.0)	6.75 (d, 7.6)	7.61 (d, 8.2)	7.58 (d, 8.2)	7.78 (d, 8.2)
13	-	-	-	-	-
14a	3.62 (d, 4.0)	4.20 (m)	2.05 (dd, 14.8,5.0)	2.02 (dd, 14.8,5.0)	2.10 (dd, 14.8, 5.2)
14b	-	-	2.58 (dd, 14.8, 9.6)	2.56 (dd, 14.8, 9.6)	2.43 (dd, 14.8, 9.0)
15	3.48 (d, 4.0)	3.92 (d, 6.2)	2.71 (m)	2.46 (m)	3.37 (t, 5.2)
16	-	-	3.06 (m)	3.04 (m)	2.76 (m)
17	1.86, 2.68 (d, 15.8)	2.72, 2.85 (d, 15.2)	4.98 (d, 8.0)	4.96 (d, 8.0)	4.86 (d, 8.0)
18	0.80 (t, 7.0)	0.72 (t, 7.2)	9.84 (s)	3.37, 3.62 (m)	1.70 (d, 7.2)
19	1.10, 1.30 (m)	0.92, 1.21 (m)	1.52 (d, 6.6)	1.48 (d, 6.6)	5.45 (q, 7.2)
20	-	-	2.87 (m)	2.07 (m)	-
21	3.63 (s)	2.80 (s)	3.96 (m)	3.71 (m)	4.03 (d, 14.2)
CO_2_*CH_3_*	3.82 (s)	3.80 (s)	2.18 (s)	2.15 (s)	2.19 (s)

*14-Chloro-15-hydroxy-vincadifformine* (**2**). colorless oil. 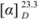
= −341.9 (*c* = 0.25, MeOH). UV (CDCl_3_) λ_max_(log*ε*) 326 (3.85), 244 (3.76), 228 (3.50), 223 (3.82), 198 (3.44) nm. IR (KBr) *ν*_max_ 3425, 1675, 1618, 1265, 1115, 760 cm^−1^. ^1^H-NMR (CDCl_3_, 600 MHz) and ^13^C-NMR (CDCl_3_, 125 MHz) data see Table 4 and Table 1 respectively. EI-MS *m/z*: 387 ([*M*−1]^+^), 389 ([*M*+1]^+^). HR-ESI-MS (pos.) *m/z*: calc. 388.1634 [M−1+H]^+^, 390.1634 [M+1+H]^+^, C_21_H_26_ClN_2_O_3_. calc. 388.1632, 390.1634).

*Perakine N_4_-oxide* (**3**). colorless oil. 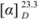
= −99.8 (*c* = 0.16, MeOH). UV (CDCl_3_) λ_max_(log*ε*) 264 (3.80), 220 (3.83) nm. IR (KBr) *ν*_max_ 3435, 2950, 1725, 1650, 1235, 1040, 765 cm^−1^. ^1^H-NMR (CDCl_3_, 600 MHz) and ^13^C-NMR (CDCl_3_, 125 MHz) data see Table 4 and Table 1 respectively. EI-MS *m/z*: 366 ([*M*]^+^). HR-ESI-MS (pos.) *m/z*: 389.1475 ([*M*+Na]^+^, C_21_H_22_N_2_O_4_Na. calc. 389.1477).

*Raucaffrinoline N_4_-oxide* (**4**). colorless oil. 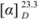
= −30.2 (*c* = 0.30, MeOH). UV (CDCl_3_) λ_max_(log*ε*) 258 (3.85), 220 (3.82) nm. IR (KBr) *ν*_max_ 3425, 2935, 1740, 1590, 1233, 1033, 750 cm^−1^. ^1^H-NMR (CDCl_3_, 600 MHz) and ^13^C-NMR (CDCl_3_, 125 MHz) data see Table 4 and Table 1 respectively. EI-MS: 368 ([*M*]^+^). HR-ESI-MS (pos.) *m/z*: 391.1635 ([*M*+Na]^+^, C_21_H_24_N_2_O_4_Na. calc. 391.1634).

*Vinorine N_1_,N_4_-dioxide* (**5**). colorless oil. 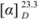
= −62.5 (*c* = 0.23, MeOH). UV (CDCl_3_) λ_max_(log*ε*) 265 (3.85), 220 (3.83), 195 (3.83) nm. IR (KBr) *ν*_max_ 3423, 2964, 1745, 1650, 1230, 1035, 753 cm^−1^. ^1^H-NMR (CDCl_3_, 600 MHz) and ^13^C-NMR (CDCl_3_, 125 MHz) data see Table 4 and Table 1 respectively. EI-MS *m/z*: 366 ([*M*]^+^). HR-ESI-MS (pos.) *m/z*: 367.1653 ([*M*+H]^+^, C_21_H_23_N_2_O_4_. calc. 367.1658).

### 3.4. Cytotoxicity Assay *in Vitro*

The isolated compounds **1**–**8** were subjected to cytotoxic evaluation against BEN-MEN-1 (meningioma), CCF-STTG1 (astrocytoma), CHG-5 (glioma), SHG-44 (glioma), U251 (glioma), SK-MEL-2 (human skin cancer), and MCF-7 cells (human breast cancer) employing the revised MTT method [34]. Adriamycin was used as the positive control. All tumor cell lines were cultured on RPMI-1640 medium supplemented with 10% fetal bovine serum, 100 U mL^−1^ penicillin and 100 μg/mL streptomycin in 25-cm^2^ culture flasks at 37 °C in humidified atmosphere with 5% CO_2_. For the cytotoxicity tests, cells in exponential growth stage were harvested from culture by trypsin digestion and centrifuging at 180 × g for 3 min, then resuspended in fresh medium at a cell density of 5 × 10^4^ cells per ml. The cell suspension was dispensed into a 96-well microplate at 100 μL per well, and incubated in humidified atmosphere with 5% CO_2_ at 37 °C for 24 h, and then treated with the compounds at various concentrations (0, 1, 10, 100 μM). After 48 h of treatment, 50 μL of 1 mg/mL MTT solution was added to each well, and further incubated for 4 h. The cells in each well were then solubilized with DMSO (100 μL for each well) and the optical density (OD) was recorded at 570 nm. All drug doses were tested in triplicate and the IC_50_ values were derived from the mean OD values of the triplicate tests versus drug concentration curves. All cell lines were purchased from the Cell Bank of the Shanghai Institute of Biochemistry & Cell Biology, Chinese Academy of Sciences (Shanghai, China).

### 3.5. Anti-inflammatory Assay *in Vitro*

The anti-inflammatory activities were determined according to a literature method with minor modifications [35]. The reaction system was incubated at 25 °C for 5 min, by sequential addition of the buffer, heme, test compounds, and Cox-1 or Cox-2 into the system followed by mixing with TMPD and arachidonic acid. The absorbance value was recorded at a wavelength of 590 nm after another 15 min of incubation at 25 °C. SC-560 and NS-398 were used as positive controls, which gave the inhibition of Cox-1 (63.20%) and Cox-2 (97.13%) respectively (Table 3). All cell lines were purchased from the Cell Bank of Shanghai Institute of Biochemistry & Cell Biology, Chinese Academy of Sciences. (Shanghai, China).

## 4. Conclusions

In summary, a chemical investigation of the 80% EtOH extract of the dried whole plant of *A. yunnanensis* led to the isolation of five new monoterpenoid indole alkaloids: 11-hydroxy-6,7-epoxy-8-oxovincadifformine (**1**), 14-chloro-15-hydroxyvincadifformine (**2**), perakine *N*_4_*-*oxide (**3**), raucaffrinoline *N*_4_*-*oxide (**4**), and vinorine *N*_1_*,N*_4_-dioxide (**5**), together with three known compounds: 11-methoxy-6,7-epoxy-8-oxo-vincadifformine (**6**), vinorine *N*_4_-oxide (**7**) and vinorine (**8**). All the alkaloids were evaluated *in vitro* for cytotoxic activities against seven tumor cell lines and anti-inflammatory properties. Alkaloids **3**, **4** and **7** showed particular cytotoxic activities against astrocytoma and glioma cell lines (CCF-STTG1, CHG-5, SHG-44 and U251) with low IC_50_ value (<20 μM) and selective inhibition of Cox-2 (>85%).
